# Long-chain omega-3 from low-trophic-level fish provides value to farmed seafood

**DOI:** 10.1002/lite.201500006

**Published:** 2015-02-19

**Authors:** Douglas M Bibus

**Affiliations:** The University of Minnesota and Lipid Technologies LLC, Lipid Technologies, LLCPO BOX 216, Austin, MN, 55912

## Abstract

Low-trophic-level fish are a crucial source of long-chain (LC) omega-3 fatty acids for farmed fish and humans. Many farm-raised fish species have a clear need for these nutrients. Farmed fish deposit the LC omega-3s in their flesh and transfer them up the food chain. However, the content of LC omega-3s in farm-raised seafood continues to decline, while the content of shorter-chain plant-sourced omega-3s, and pro-inflammtory omega-6s continue to increase. This reduces its nutritional worth. The value of low-trophic-level fish is often viewed merely as its price at the dock. Some reports and metrics steer public attention towards the mass balance between quantities of low-trophic-level fish and farmed seafood. However, the the nutritional value of seafood is more important than its mere quantities. The role of low-trophic-level fish in human nutrition, health, and wellbeing is a fundamental component of its economic value to society.

## Introduction

Global aquaculture production has increased for several decades and further growth is expected. The supply of fishmeal and fish oil has been relatively constant and is not expected to increase. The inclusion rates of these ingredients in farmed fish diets continue to decrease 1.

There are two practical sources of long chain (LC) omega-3s in today's market place – fish oil and algal oil. The annual production of fish oil is roughly one million tons, while the annual production of algal oil is a few thousand tons. Currently, algal oil production cost is one to two orders of magnitude greater than that of fish oil. Hence, it is mainly a material for niche markets and high-end products, like infant formula. Other sources now exist, perhaps most noteably GMO oilseeds and yeasts, but they are not yet widely available on the open market.

Researchers have worked for decades to replace fishmeal and fish oil with land-based ingredients. Recently, a great amount of effort has been spent looking into the mass balance between marine feed fish and aquaculture biomass 2. Lost in all this have been the issues of nutritional value and public health. Many have chosen to highlight the importance of reducing the inclusion of fishmeal and fish oil in aquaculture diets as an attribute of “sustainability” 2. The message has been clear – less fish used is better. However, there are many sustainable fisheries used for fishmeal and fish oil, and, the use of these ingredients is not in and of itself “unsustainable” 3. Further, it has been reported that the environmental footprint of fishmeal and fish oil production from small pelagic species, like Gulf menhaden, is much smaller than that of terrestrial aquafeed ingredients like wheat gluten meal 4.

Yet, some consumers are now purchasing seafood that is raised on feeds made predominantly from terrestrial ingredients, because they believe it to be more “sustainable” and assume it provides all the benefits of seafood. But this seafood oftentimes has less health benefits than wild-caught or farm-raised seafood that was raised with more marine ingredients 5. Because many consumers purchase farm-raised seafood for the health benefits derived from feeding fishmeal and fish oil to fish, the industry needs to be cautious with messaging, or risk losing health-conscious consumers.

## Omega-3s in fish diets and human health

The LC omega-3s, eicosapentaenoic acid (EPA), docosapentaenoic acid (DPA) and docosahexaenoic acid (DHA) come from marine sources. Shorter-chain omega-3s, like α-linolenic acid (ALA), are more prevalent in terrestrial plants. The LC omega-3s are the precursors for anti-inflammatory mediators and serve as building blocks for neural tissues. The shorter chain counterparts are inefficiently elongated.

The total omega-3 content of farmed fish varies greatly 6, as the fatty acid profile of fish mirrors that of their diet 1,5. In 2004, farmed salmon contained three to four times more omega-3s than omega-6s 7. By 2014, the omega-3 to omega-6 ratio has decreased, and in some cases has even reversed 1,8. Wild salmon contains approximately ten times more omega-3s than omega-6s 8. It also contains one half to one fifth fillet fat as compared to farmed salmon 6. The stable natural ratio of these fatty acids in wild salmon is expected, as they are virtually unexposed to land-based sources of fat.

It is true that modern-day farmed salmon remains a good source of total omega-3s. However, it typically provides only 34% less saturated fat than hamburger meat. The total fat content of farmed salmon masks its declining LC omega-3 content. Additionally, the composition of total omega-3s of farmed salmon is different from that of the wild. While the ALA content of the wild fish is below one percent, its current content in farm-raised salmon is only slightly below that of DHA and higher than that of EPA 8. This is a result of the diet containing terrestrial-derived omega-3s, such as ALA from rapeseed oil 8. The potential impact of this shift on cosumer health is yet to be determined.

It is worth noting that not all fish must be excellent sources of omega-3s 8. Tilapia, for example, has very little fillet fat content and is a good source of high-quality protein 8.  Feeding high fish-oil diets to tilapia is not cost- or resource-efficient, as little fat is deposited in the edible portion 8.  However, oily fish, such as salmon, are often consumed specifically because of its nutritional value attributable to LC omega-3s 8.  Therefore, farm-raised oily fish should be fed LC omega-3s, so consumers realize the benefits they expect 3,8.  

Poor diet and lack of essential nutrients is recognized as one of the leading causes of preventable diseases in the United States. The American Heart Association recommends that people consume oily fish twice per week. Similarly, the World Health Organization, the national health agencies of numerous European countries, Australia and Japan recommend the consumption of oily fish or omega-3 fatty acids. But again, oily fish differ in fat composition, which result in different effects on health.

A Norwegian study determined the impact of substitution of fish oil in salmon diets with rapeseed oil on markers of cardiovascular health in consumers 5. In that study, the salmon had been raised on diets formulated with 100% fish oil, 100% rapeseed oil or the equal blend 5. As expected, the lipid profiles of salmon mirrored those of the diets they consumed 5. The omega-3 to omega-6 ratio of fish fed the 100% rapeseed oil diet was less than one sixteenth that of the fish fed the 100% fish oil diet, and less than one third of that that of fish fed a mix of rapeseed and fish oils 5. Also, as expected, consumption of salmon raised on the 100% fish oil diet led to reduction of serum triglycerides and enacted other favorable biochemical changes in the patients 5. In the other two groups, these effects were insignificant 5. If the LC omega-3 content in fish diets continues to decline, leading to further decline of omega-3s in fish fillets, the AHA will need to revise its recommendations, or at least specify types of fish that are expected to beneficially affect heart health.

In addition to heart health, LC omega-3s are important in neural development, maintenance and function. Brain, retina, and other neural tissues are rich in DHA. Because of the critical role of DHA in infant development, and because elongation of shorter chain plant-based omega-3s to DHA is limited, DHA should be consumed during pregnancy and early stages of human development. Despite the importance of LC omega-3s for human health, it is often overlooked by those discussing the issue of feeding fish to fish.

## Fish in, fish out

Recently, Byelashov and Griffin 3 demonstrated that the Fish In, Fish Out (FIFO) metric, as originally described, and which is often perceived as an indicator of sustainability, is flawed. The original FIFO concept was intended to estimate the number of units of low-trophic-level fish required to support the production of one unit of particular farmed species, like salmon 2. For example, Whole Foods, the largest natural and organic grocery retailer in the U.S., requires that suppliers of farm-raised salmon provide “Annual reporting on progress towards meeting maximum FIFO ratio of 1 : 1”. This same retailer has no similar requirement for wild-caught salmon, as nature cannot meet such demands. Condensed fish solubles, a byproduct of fishmeal and fish oil production, are used as an organic fertilizer by the producers of organic fruits and vegetables, which are popular among the same consumers who demand farm-raised salmon with a “low” FIFO ratio.

Salmon diets use proportionally more fish oil and less fishmeal than can be derived from one unit of most feed fish species, like anchovy 3. Thus, the FIFO logic was built on a false premise that unused fishmeal is wasted 3. Similarly, shrimp diets use proportionally more fishmeal and less fish oil than can be derived from one unit of most feed fish species 3. In this case, the FIFO approach assumes that the unused fish oil is lost 3.

In reality, no fishmeal or fish oil goes to waste, and FIFO needs to be looked at in the aggregate 3. Previously, it was demonstrated that simply combining salmon and shrimp production resulted in a drastic change in the FIFO ratio 9. Whereas the original salmon and shrimp FIFO estimates were 4.9 and 1.4, respectively, the combined estimate was 1.7 9. Therefore, the numerator of the ratio should include quantities of low-trophic-level fish destined for fishmeal and fish oil that is used in aquaculture 3. The denominator should include the global quantities of farmed marine species that use these ingredients 3. Additionally, about 35% of the global fishmeal supply comes from fish by-products 10. This must be considered in the FIFO discussion 3. Further, Byelashov and Griffin 3 rightfully pointed out that “fish in produces a variety of outs: fish out, pets out, plants out, zoo animals out, laboratory animals out, fish oil supplements out, pharmaceuticals out, etc.” 3.

It is important to note that as global aquaculture production increases, while the supply of fishmeal and fish oil remains flat, the ratio has to decrease. The quantity of globally-produced farmed fish and shrimp in 2012 was three times higher than the quantity of fish harvested for fishmeal and fish oil 11. Thus, aquaculture and its wide spread use of terrestrial ingredients clearly results in a net increase of fish biomass to help feed the world's inhabitants 3.

Oftentimes a rough FIFO estimate of 10 : 1 is used for wild piscivorous fish based on the stair step up from one trophic level to the next. Many carnivorous fish are likely well above 10 : 1 in nature, as they are more than one trophic level above the aggregate of their food items. In one report, Yellowfin Tuna consumed 34 kilograms of feed fish to gain one kg of biomass 12. Regardless, most farmed fish have much lower FIFO than their wild cousins.

## Primary production required

The Primary Production Required (PPR) is another tool to measure the efficiency with which energy is converted from prey to predator 13. It is a function of the trophic level, which is the position of marine organisms and their food items within the food web 13. The metric represents the number of weight units of biomass that is needed to support the production of one weight unit of a particular species. High-trophic-level predators may require hundreds of times more marine resources than lower-trophic-level fish.

For instance, the trophic level of Gulf Menhaden is 2.2 and its PPR is 15.5 13,14. In other words, the production of one ton of Gulf Menhaden biomass in nature requires 15.5 tons of primary production. Peruvian anchovy has a trophic level of 2.7 and its PPR is 50.1 13,14.

**Figure 1:**
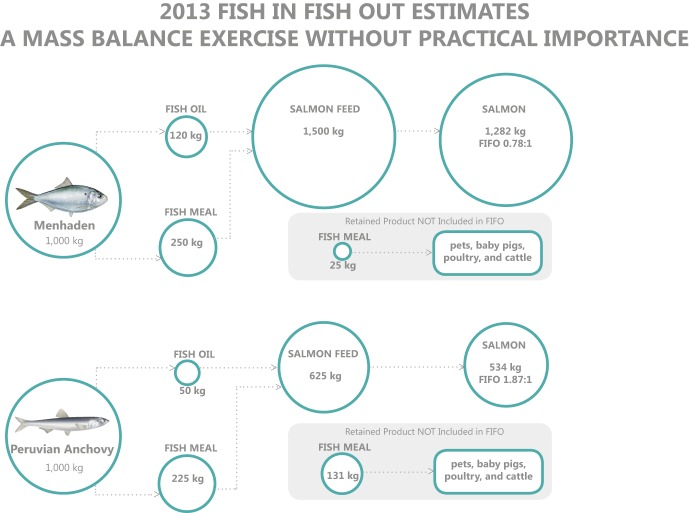
2013 Fish in fish out estimates – a mass balance exercise without practical importance. Assumptions: Gulf Menhaden oil yield, 12% 16; Gulf Menhaden fishmeal yield, 25% 16; Peruvian Anchovy oil yield, 5% 17; Peruvian Anchovy fishmeal yield, 22.5% 17; 2013 fishmeal use in salmon diets of Chilean producers, 15% 18; 2013 fish oil use in salmon diets of Chilean producers, 8% 18; Feed conversion ratio, 1.17 18.

Although the FIFO concept is flawed, as the inclusion rates of fishmeal and fish oil in salmon diets continue to decline, the theoretical calculations show that if we were to use Gulf Menhaden meal and oil only for production of Chilean Atlantic Salmon, it could have taken 0.78 units of low-trophic-level fish to produce one unit of salmon (**Figure** 1). Additionally, the remaining fishmeal from each ton of menhaden is used in diets of other farmed fish, shrimp, pets, and other animals 3,8. The author wishes to stress that this is not a reflection of reality, but simply a theoretical calculation demonstrating a bias with the opposite effect of that typically used in FIFO calculations. Thus, the theoretical PPR for farmed salmon fed diets containing menhaden fishmeal and fish oil could be 12.1 (i.e. 15.5 × 0.78).

Although there may be some differences in the efficiencies of conversion of fishmeal and fish oil derived from small pelagic species into carnivorous fish species, this conversion is far superior to the efficiency of conversion in nature. It is true that farmed fish do not have to expend as much energy to eat a prepared diet as wild fish expend hunting down prey. However, the primary reason is because meal and oil from small pelagic fish is significantly subsidized by land-based production, like rapeseed oil and soybean meal. As wild Atlantic Salmon has a PPR of 2691.5 14, in terms of marine resources used, the theoretical conversion of menhaden into farmed salmon is 222.4 (i.e. 2691.5/12.1) times more efficient than the production of salmon biomass in nature.

## Value of fish

Recently, the Lenfest Forage Fish Taskforce published “A Little Fish Big Impact” 15. It stated that low-trophic-level fish are two times more valuable when left in the sea than when harvested 15. But this logic is incomplete. To increase predator biomass, the stocks of predator fish would have to be food-limited. This is not a valid assumption for all stocks. Simply increasing food supply will not appreciably increase many predator stocks in the marine environment.

Another issue with the contention that low-trophic-level fish should not be harvested is that it compares the actual value of low-trophic-level fish to the value of theoretical predator fish. The comparison should be between the value of the actual products derived from low-trophic-level fish (farmed fish and shrimp, pharmaceuticals, human and pet nutrition products, etc.), to the value of theoretical products (predator fish and possibly other animals) derived from low-trophic-level fish. This analysis has a very different result, which is far more favorable to the use of fishmeal and fish oil derived from low-trophic-level fish.

Further, if predator biomass did increase, “economic value” can only be realized through increased harvest of predator fish. But, obtaining marine nutrients through predators is much less efficient than getting them from prey fish. As we move from one trophic level to the next, only about 10% of the nutrients are retained, while the rest are undigested, used in metabolic pathways or burned as energy. Because of this, harvesting wild higher-trophic level carnivorous fish is a much less efficient way of providing marine-derived essential nutrients, particularly LC omega-3s, to humans.

Also, some bony and oily fish, like menhaden, are not typically used for direct human consumption. Therefore, unless someone finds a way to cost-effectively harvest marine phytoplankton for omega-3s, or consumers develop a taste for bony small fish, fishmeal and fish oil will remain the practical way to deliver the essential nutrients from the ocean to humans for their health and wellbeing.

In many cases, if left at sea, the fish biomass will be wasted in terms of being a source of LC omega-3s for humans. This resource should not go to waste, when people are suffering from a range of diseases, which may be prevented, delayed, or eased by appropriate diet and adequate consumption of marine omega-3s. The value of human health must be considered.

## Conclusion

However interesting these metrics are, they distract the public from the health value of fish. The policies of some retailers and producers – to reduce FIFO – directly lead to the displacement of marine LC omega-3s with the shorter-chain plant-sourced omega-3s in fish filets. Concurrently, the content of pro-inflammatory omega-6s in farm-raised seafood continues to increase. This is an unintended consequence of replacing marine ingredients with land-based ingredients, which are perceived as more sustainable. Because reputable salmon feed producers only use responsibly sourced ingredients, this is a misguided policy with negative unintended consequences.
